# miR-33-5p, a novel mechano-sensitive microRNA promotes osteoblast differentiation by targeting Hmga2

**DOI:** 10.1038/srep23170

**Published:** 2016-03-16

**Authors:** Han Wang, Zhongyang Sun, Yixuan Wang, Zebing Hu, Hua Zhou, Lianchang Zhang, Bo Hong, Shu Zhang, Xinsheng Cao

**Affiliations:** 1 The Key Laboratory of Aerospace Medicine, Ministry of Education, The Fourth Military Medical University, 710032, Xi’an, Shaanxi, China; 2 Department of orthopedics, No. 454 Hospital of PLA, 210002, Nanjing, Jiangsu, China

## Abstract

MicroRNAs (miRNAs) interfere with the translation of specific target mRNAs and are thought to thereby regulate many cellular processes. However, the role of miRNAs in osteoblast mechanotransduction remains to be defined. In this study, we investigated the ability of a miRNA to respond to different mechanical environments and regulate mechano-induced osteoblast differentiation. First, we demonstrated that miR-33-5p expressed by osteoblasts is sensitive to multiple mechanical environments, microgravity and fluid shear stress. We then confirmed the ability of miR-33-5p to promote osteoblast differentiation. Microgravity or fluid shear stress influences osteoblast differentiation partially via miR-33-5p. Through bioinformatics analysis and a luciferase assay, we subsequently confirmed that Hmga2 is a target gene of miR-33-5p that negatively regulates osteoblast differentiation. Moreover, miR-33-5p regulates osteoblast differentiation partially via Hmga2. In summary, our findings demonstrate that miR-33-5p is a novel mechano-sensitive miRNA that can promote osteoblast differentiation and participate in the regulation of differentiation induced by changes in the mechanical environment, suggesting this miRNA as a potential target for the treatment of pathological bone loss.

As cells respond to mechanical stimulation, bone tissue adjusts via a feedback system. Specifically, changes in the local mechanical environment signal bone cells to modify bone structure to meet the new requirements^1^,^2^. Mechanical unloading causes a marked loss of bone due to the impairment of bone formation, whereas mechanical loading that results in high strains increases bone formation^3^,^4^. These changes ultimately affect bone formation and resorption by affecting the balance between osteoblast and osteoclast activity^5^,^6^. Normally, almost the entire bone matrix is mineralized by osteoblasts, but only mature osteoblasts can accomplish this important work. Therefore, osteoblast differentiation is a critical process in bone formation. Additionally, osteoblast differentiation is triggered by mechanical stimulation via the secretion of hormones and growth factors^7^,^8^,^9^.

Four main types of mechanical loading systems are used to simulate the mechanical loading environment of osteoblasts in bone tissue: fluid shear stress (FSS), hydrostatic compression, biaxial stretching, and uniaxial stretching on cultured osteoblastic cells^10^. FSS is a common type of mechanical stimulation in bone. Increasingly strong evidence suggests that fluid shear is one of the principal forces responsible for bone adaptation. Weight bearing and locomotion stimulate the flow of interstitial fluid through the bone canalicular system, and the resultant shear stress is thought to be a major mechanism whereby mechanical forces stimulate bone growth^10^,^11^. Shear stress activates various signal transduction pathways and initiates an anabolic response in osteoblasts, which leads to changes in gene expression and increased cell differentiation^12^. Conversely, mechanical unloading due to prolonged bed rest, immobilization, or microgravity in space causes a marked loss of bone due to a significant imbalance between bone formation and resorption, and the impairment of bone formation is partially due to the inhibition of osteoblast differentiation^13^,^14^. Interestingly, unlike FSS and stretching, which are contact forces, gravity is a non-contact force. Dramatic decreases in gravity, such as microgravity, lead to decreased osteoblastogenesis and increased adipogenesis in human mesenchymal stem cells (hMSC) via the disruption of stress fibers, altered integrin signaling and down-regulated expression of well-known markers and regulators of osteoblast differentiation, including osteocalcin (OC), type I collagen α1 (Col-Iα1), dentin matrix protein 1 (DMP1) and runt-related transcription factor 2 (Runx2)^15^,^16^. Moreover, the secretion and calcification of the osteoid requires the participation of mature osteoblasts. Thus, the inhibition of osteoblast differentiation suppresses osteoblast mineralization and eventually affects bone formation. Therefore, elucidating the mechanisms through which contact forces enhance osteoblast differentiation and non-contact forces, such as an unloading, reduce this differentiation is important.

MicroRNAs (miRNAs) are endogenously expressed non-coding single-stranded RNAs of 20–24 nucleotides that post-transcriptionally regulate gene expression. Their regulatory functions are widespread in various biological processes^17^,^18^. Recent studies have discovered multiple microRNAs that are important regulators of bone-forming genes, including essential transcription factors as well as developmental signaling molecules and their receptors, which are required for the complex process of osteoblastogenesis. miR-133 and miR-135 functionally inhibit the differentiation of osteoprogenitors by attenuating the Runx2 and Smad5 pathways, which synergistically contribute to bone formation^19^. Furthermore, miR-218 enhances Wnt activity and regulates osteoblastic genes that contribute to the homing and growth of metastatic cells to bone^20^. miR-210 ameliorates postmenopausal osteoporosis due to estrogen deficiency by promoting VEGF expression and osteoblast differentiation^21^. These findings demonstrate that miRNAs significantly impact osteoblast differentiation.

In addition, previous research indicated that some miRNAs are sensitive to mechanical stimuli, which could regulate the function of osteoblasts. For example, some miRNAs are involved in FSS-induced pre-osteoblast differentiation, such as miR-20a, miR-21, miR-19b, miR-34a, miR-34c, miR-140, and miR-200b^22^. miR-103 is up-regulated in response to cyclic mechanical stretch-induced osteoblast differentiation and markedly inhibits osteoblast differentiation^23^. Moreover, miR-153 is sensitive to mechanical loading and regulates osteoblast differentiation by directly targeting BMPR2^24^. These results demonstrate that miRNAs may play a critical role in the sensing of mechanical loads by osteoblasts. Moreover, our previous work showed that simulated microgravity can up-regulate the expression of miR-103, resulting in down-regulation of Cav1.2 expression, inhibition of LTCC function and inhibition of osteoblast proliferation^25^,^26^. However, to our knowledge, miRNAs have not been shown to be sensitive to two different mechanical environments: a contact force environment and a non-contact force environment. Therefore, the response of miRNAs to different mechanical environments and their effect on osteoblast differentiation warrant an investigation.

In this study, we identified miR-33-5p as a mechano-sensitive miRNA whose expression can significantly be altered in response to FSS or a simulated microgravity environment. Furthermore, miR-33-5p expression positively correlated with osteoblast differentiation. Specifically, miR-33-5p promotes osteoblast differentiation by directly targeting the 3′ UTR of Hmga2. Although Hmga2 has been proven to play an important role in cancer^27^, we confirmed its regulatory effect on osteoblast differentiation. This study may provide a novel mechanism and potential therapeutic target for skeletal disorders caused by pathological mechanical environments.

## Results

### miR-33-5p levels in MC3T3-E1 cells are altered under different mechanical environments

**Figure 1:**
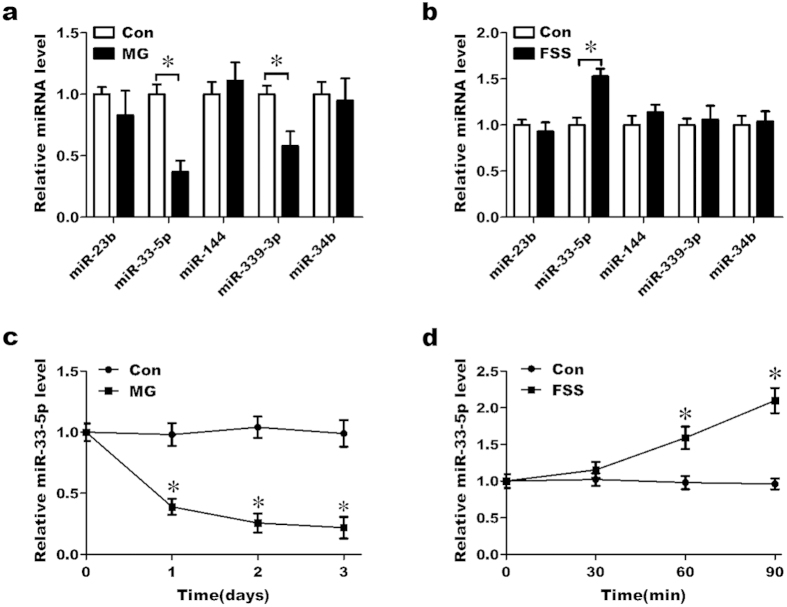
miR-33-5p levels are altered under different mechanical environments in MC3T3-E1 cells. (**a**) qRT-PCR analysis of the expression of 5 selected miRNAs in MC3T3-E1 cells in response to simulated microgravity for 2 d. (**b**) qRT-PCR analysis of the expression of 5 selected miRNAs in MC3T3-E1 cells treated with fluid shear stress (FSS) (10 dynes/cm^2^) for 1 h. (**c**) TaqMan miRNA assay of the expression pattern of miR-33-5p in MC3T3-E1 cells in response to simulated microgravity for 3 d. (**d**) TaqMan miRNA assay of the expression pattern of miR-33-5p in MC3T3-E1 cells treated with FSS (10 dynes/cm^2^) for 90 min. The values are shown relative to those before treatment. The data are expressed as the mean ± SD of three replicates each. *P < 0.05 vs. the control.

To investigate the changes in miRNA expression in response to different mechanical environments, MC3T3-E1 cells were treated with simulated microgravity for 2 days or with 10 dynes/cm^2^ FSS for 1 h. miRNA was then extracted for quantitative real-time PCR (qRT-PCR) analysis. Based on the miRNA microarray data of our previous study, miRNA-339-3p, -33-5p, -34b, -23b, -144 were selected to be the candidate miRNAs. qRT-PCR testing of 5 selected miRNAs showed that the expression of miR-33-5p and miR-339-3p was down-regulated in response to a simulated microgravity environment (Fig. 1a), whereas the expression of miR-33-5p was up-regulated by FSS (Fig. 1b). Significantly, miR-33-5p showed contrasting expression patterns under different mechanical environments. Therefore, we focused on the role of miR-33-5p and further examined the changes in its expression over time in response to a simulated microgravity environment or 10 dynes/cm^2^ FSS by the method of TaqMan miRNA assay and qRT-PCR. The results of TaqMan miRNA assay showed that, compared with the corresponding control group, the expression level of miR-33-5p continuously decreased over time in response to simulated microgravity (Fig. 1c) and increased starting at 60 min of FSS until 90 min of FSS (Fig. 1d). And the results of qRT-PCR showed similar alterations in the expression level of miR-33-5p in response to simulated microgravity (Supplementary Fig. 1a) and to 90 min of FSS (Supplementary Fig. 1b).

### miR-33-5p promotes the differentiation of MC3T3-E1 cells

**Figure 2:**
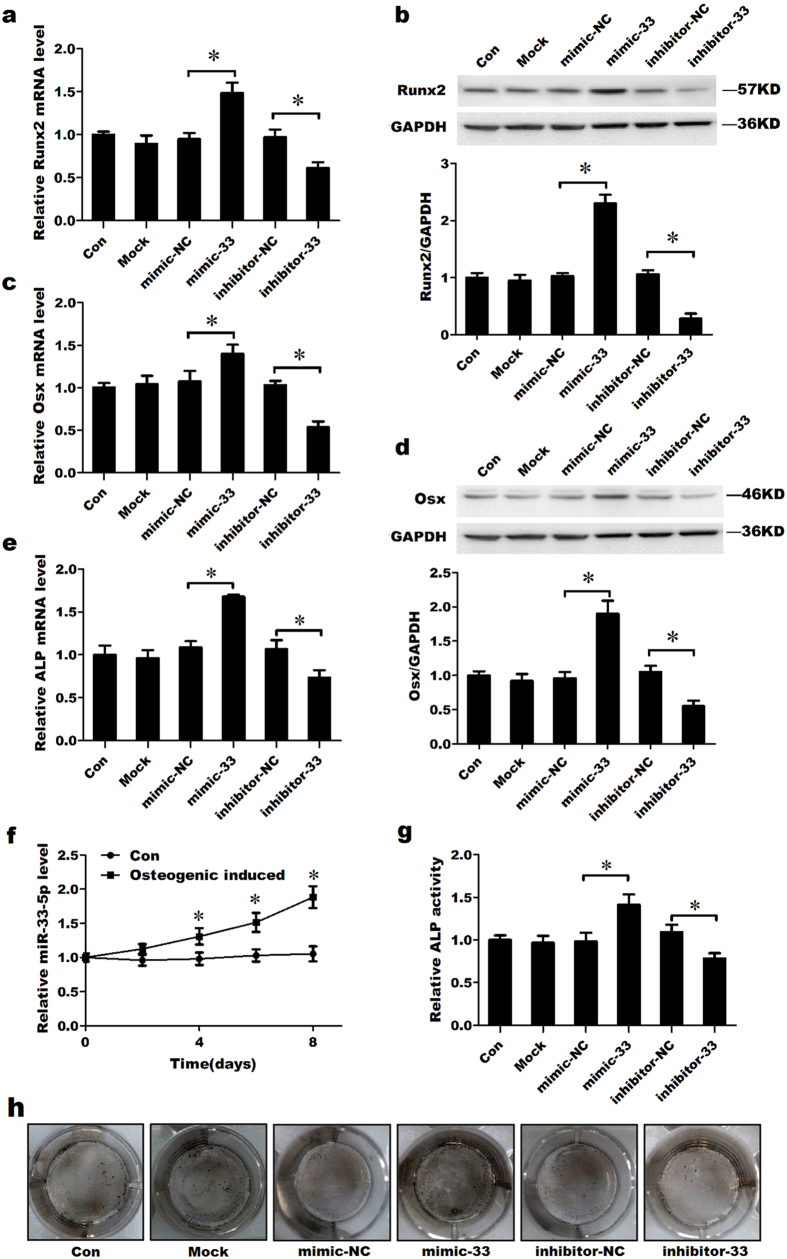
miR-33-5p promotes the differentiation of MC3T3-E1 cells. (**a**,**c**,**e**) qRT-PCR analysis of the changes in the mRNA expression levels of Runx2, Osx and ALP in MC3T3-E1 cells after treatment with mimic-33, inhibitor-33 or their negative controls for 48 h. (**b**,**d**) Western blot analysis of the changes in Runx2 and Osx protein levels in MC3T3-E1 cells after treatment with mimic-33, inhibitor-33 or their negative controls for 48 h. (**f**) TaqMan miRNA assay of the expression pattern of miR-33-5p during the differentiation of MC3T3-E1 cells. (**g**) The activity analysis of ALP in MC3T3-E1 cells after treatment with mimic-33, inhibitor-33 or their negative controls for 48 h. (**h**) Representative images of ALP staining in MC3T3-E1 cells after treated with mimic-33, inhibitor-33 or their controls. The values are shown relative to those before treatment. The Western blot gels were cropped and the full-length gels are presented in Supplementary Fig. 6. The data are expressed as the mean ± SD of three replicates each. *P < 0.05 vs. the control.

To examine the role of miR-33-5p in the osteogenic differentiation of MC3T3-E1 cells, we used a miR-33-5p mimic to overexpress miR-33-5p or a miR-33-5p inhibitor to knock down the expression of miR-33-5p in MC3T3-E1 cells. The maximum dosage of 100 nM was used for the transfection of inhibitor-33. Inhibitor-33 suppressed the expression of this miRNA by approximately 50%, whereas transfection with mimic-33 resulted in a nearly 70-fold increase in miR-33-5p expression 48 h after transfection (Supplementary Fig. 2). The effects of miR-33-5p on the osteogenic differentiation of MC3T3-E1 cells were investigated by observing the expression levels of the osteoblast-specific markers Runx2, Osx and ALP. The qRT-PCR results showed that the mRNA expression levels of Runx2, Osx and ALP significantly increased following transfection with mimic-33, whereas they decreased markedly following transfection with inhibitor-33 compared with the transfection of mimic-33-NC or inhibitor-33-NC, respectively (Fig. 2a,c,e). Accordingly, the protein expression levels of Runx2 and Osx significantly increased following transfection with mimic-33 and decreased markedly following transfection with inhibitor-33 compared with the transfection of mimic-33-NC or inhibitor-33-NC, respectively (Fig. 2b,d). ALP activity and ALP staining remarkably enhanced in the mimic-33 treatment group, whereas the activity significantly decreased in the inhibitor-33 treatment group, as compared with corresponding control group (Fig. 2g,h). To further verify the role of miR-33-5p in osteogenic differentiation, the expression levels of miR-33-5p during osteogenic differentiation were evaluated by TaqMan miRNA assay and qRT-PCR analysis. The results of TaqMan assay showed that miR-33-5p expression was notably increased starting from the fourth day after the induction of osteogenic differentiation in MC3T3-E1 cells (Fig. 2f). And the result of qRT-PCR showed similar alteration in the expression level of miR-33-5p after the induction of osteogenic differentiation (Supplementary Fig. 1c).

### miR-33-5p partially attenuates the inhibition of MC3T3-E1 differentiation by simulated microgravity

**Figure 3:**
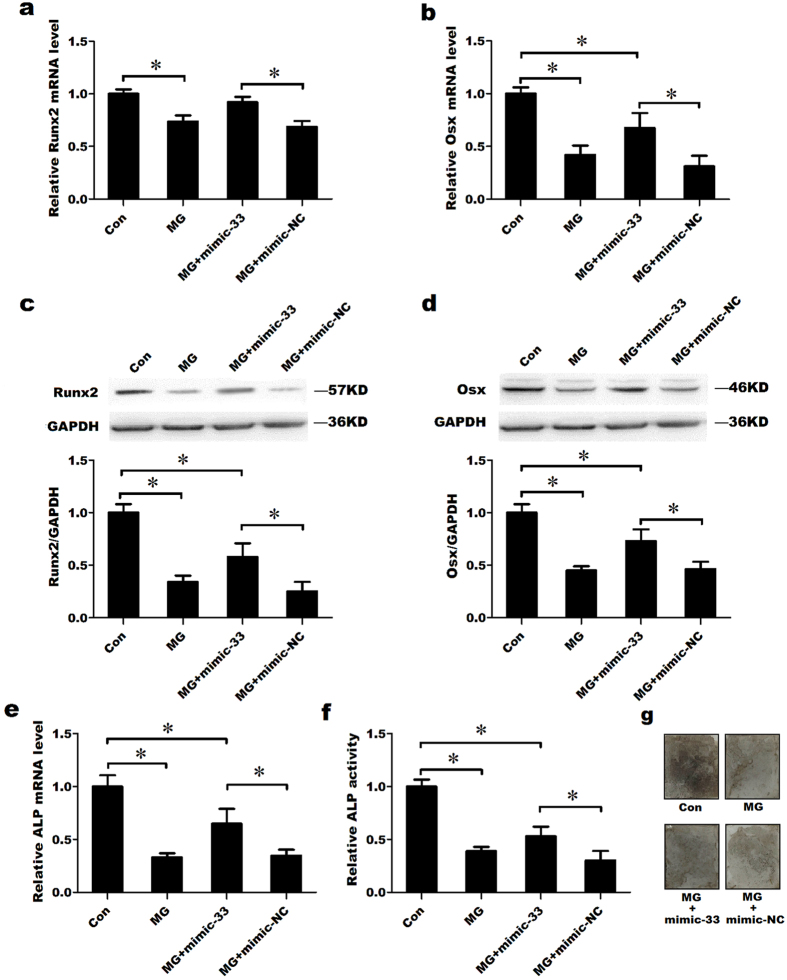
miR-33-5p partially attenuates the inhibition of MC3T3-E1 differentiation by simulated microgravity. (**a**,**b**,**e**) qRT-PCR analysis of changes in the mRNA expression of Runx2, Osx and ALP in MC3T3-E1 cells after treatment with mimic-33 and its negative control in a simulated microgravity environment for 48 h. (**c**,**d**) Western blot analysis of the changes in Runx2 and Osx proteins levels in MC3T3-E1 cells after treatment with mimic-33 or its negative control in a simulated microgravity environment for 48 h. (**f**) The activity analysis of ALP in MC3T3-E1 cells after treatment with mimic-33 or its negative control in a simulated microgravity environment for 48 h. (**g**) Representative images of ALP staining in MC3T3-E1 cells after treated with mimic-33 or its negative control in a simulated microgravity environment for 48 h (the cells were seeded on coverslips). The values are shown relative to those before treatment. The Western blot gels were cropped and the full-length gels are presented in Supplementary Fig. 6. Data are expressed as the mean ± SD of three replicates each. *P < 0.05 vs. the control.

We confirmed that miR-33-5p was down-regulated in MC3T3-E1 cells in response to a simulated microgravity environment. Many previous studies have shown that the osteogenic differentiation of osteoblasts is suppressed in response to a simulated microgravity environment. Therefore, to study the role of miR-33-5p in the inhibition of osteoblast differentiation by simulated microgravity, mimic-33 and its negative control were transfected into MC3T3-E1 cells prior to subjecting the cells to clinorotation for 48 h. Total RNA and proteins were then extracted for PCR and western blot tests. The qRT-PCR analysis showed that simulated microgravity significantly down-regulated the mRNA levels of Runx2 and Osx compared with the control group, which was cultured in a normal gravity environment. The overexpression of miR-33-5p significantly attenuated the decreases in Runx2, Osx and ALP mRNA levels induced by simulated microgravity (Fig. 3a,b,e). However, Osx mRNA expression was not restored to control levels. Similar results were observed at the protein level, where western blotting showed that the transfection of mimic-33 rescued the protein expression of Runx2 and Osx in a simulated microgravity environment compared with the NC group, but these levels were not restored to control levels (Fig. 3c,d). Moreover, similar trend was observed in the ALP activity assay and ALP staining, transfection of mimic-33 partially rescued the ALP activity and ALP staining in a simulated microgravity environment compared with the NC group (Fig. 3f,g).

### Knockdown of miR-33-5p partially inhibits the FSS-induced differentiation of MC3T3-E1 cells

**Figure 4:**
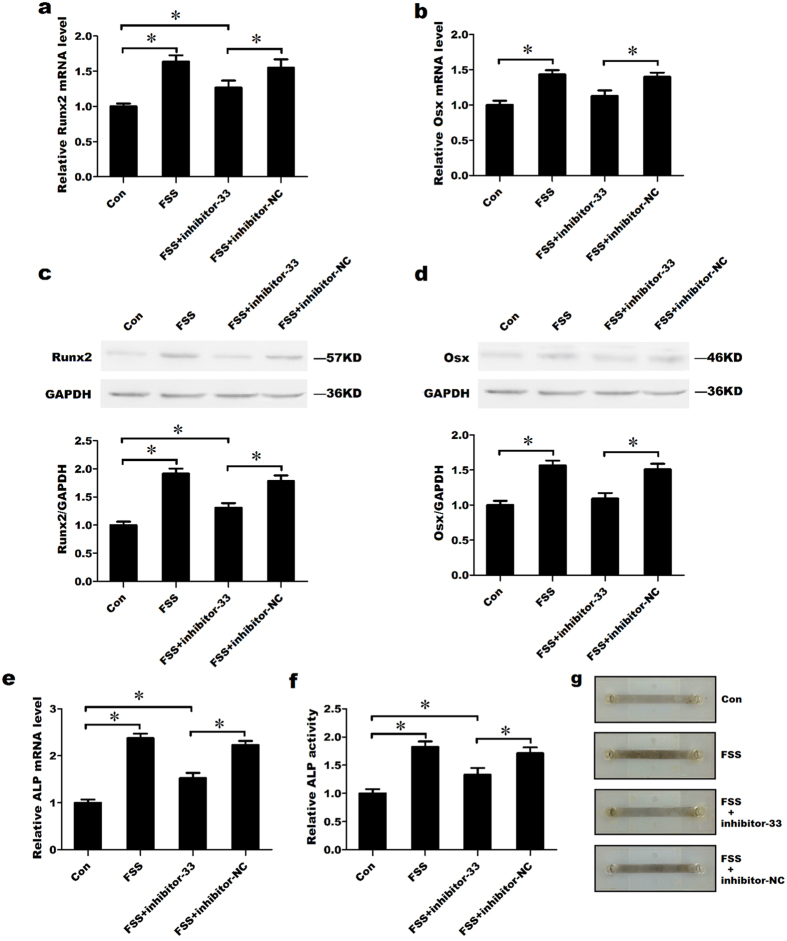
Knockdown of miR-33-5p partially inhibits the FSS-induced differentiation of MC3T3-E1 cells. (**a**,**b**,**e**) qRT-PCR analysis of the changes in the mRNA expression of Runx2, Osx and ALP in MC3T3-E1 cells after treatment with inhibitor-33 or its negative control under FSS environment (10 dynes/cm^2^) for 1 h. (**c**,**d**) Western blot analysis of the changes in Runx2 and Osx proteins levels in MC3T3-E1 cells after treatment with inhibitor-33 or its negative control under FSS environment (10 dynes/cm^2^) for 1 h. (**f**) The activity analysis of ALP in MC3T3-E1 cells after treatment with inhibitor-33 or its negative control under FSS environment (10 dynes/cm^2^) for 1 h. (**g**) Representative images of ALP staining in MC3T3-E1 cells after treated with inhibitor-33 or its negative control under FSS environment (10 dynes/cm^2^) for 1 h (the cells were seeded in FSS culture slides, detailed information of slide is in supplementary Fig. 5). The values are shown relative to those before treatment. The Western blot gels were cropped and the full-length gels are presented in Supplementary Fig. 6. The data are expressed as the mean ± SD of three replicates each. *P < 0.05 vs. the control.

Many studies have shown that FSS stimulation can promote the differentiation of osteoblasts. We found that FSS up-regulated miR-33-5p in MC3T3-E1 cells. Therefore, to further investigate the role of miR-33-5p in FSS-induced osteoblast differentiation, inhibitor-33 and its negative control were transfected into MC3T3-E1 cells before treating the cells with FSS for 1 h; total RNA and proteins were then extracted for PCR and western blot tests. The qRT-PCR analysis showed that FSS markedly up-regulated the mRNA levels of Runx2 and Osx compared with the control group. The knockdown of miR-33-5p significantly attenuated the increases in Runx2, Osx and ALP mRNA expression induced by FSS (Fig. 4a,b,e). Similar results were observed at the protein level, where the transfection of inhibitor-33 significantly decreased the protein expression of Runx2 and Osx in response to FSS compared with the NC group, as indicated by western blotting. Again, the protein expression of Runx2 was not restored to control level (Fig. 4c,d). Furthermore, transfection of inhibitor-33 partially decreased ALP activity and ALP staining in response to FSS compared with the NC group (Fig. 4f,g).

### Hmga2 is the target gene of miR-33-5p in MC3T3-E1 cells

To further explore the mechanism by which miR-33-5p regulates osteoblast differentiation, a bioinformatics analysis was performed using targetScan, miRanda and miRBase miRNA target prediction software to screen for target genes of miR-33-5p. Based on these analyses, Hmga2 received the highest composite score.

**Figure 5:**
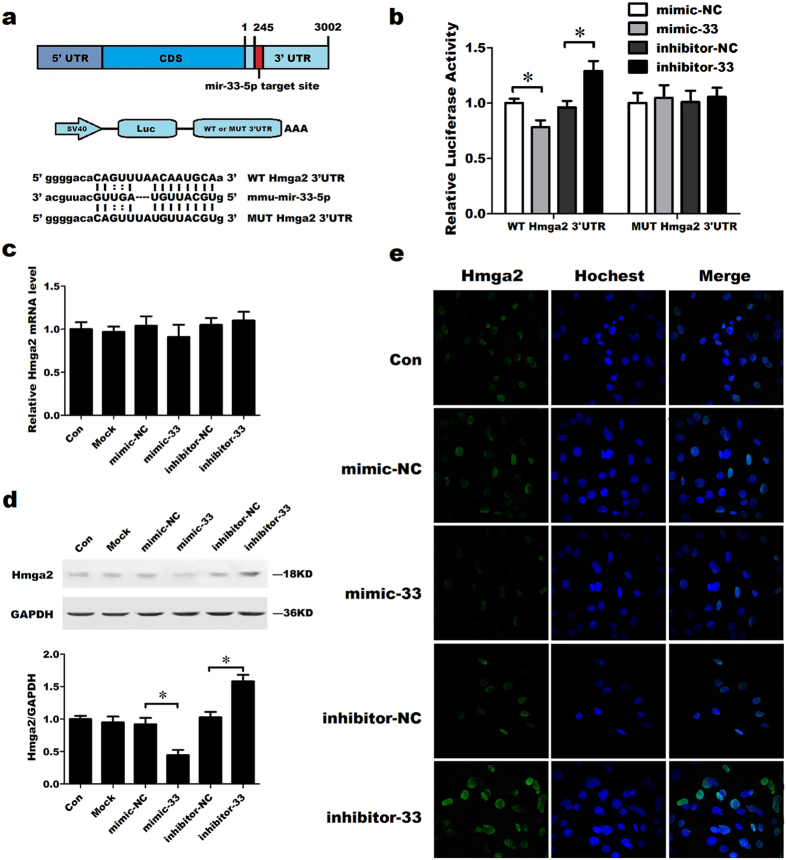
Hmga2 is the target gene of miR-33-5p in MC3T3-E1 cells. (**a**) A schematic illustration of the design of luciferase reporters containing the WT Hmga2 3′ UTR (WT 3′ UTR) or the site-directed mutant Hmga2 3′ UTR (MUT 3′ UTR). (**b**) The effects of the miR-33-5p mimic and inhibitor or their negative controls on the luciferase activity of the WT Hmga2 3′ UTR or MUT Hmga2 3′ UTR reporter in MC3T3-E1 cells. The values in the condition of WT Hmga2 3′ UTR or MUT Hmga2 3′ UTR shown relative to that of the mimic-NC in the same condition. (**c**) qRT-PCR analysis of the changes in the mRNA expression of Hmga2 in MC3T3-E1 cells after treatment with mimic-33, inhibitor-33 or their negative controls. (**d**) Western blot analysis of the changes in Hmga2 proteins levels in MC3T3-E1 cells after treatment with mimic-33, inhibitor-33 or their negative controls for 48 h. (**e**) Immunostaining analysis of the changes in Hmga2 protein expression after treatment with mimic-33, inhibitor-33 or their negative controls for 48 h. Green: Hmga2, blue: Hoechst staining of nuclei. All photomicrographs were recorded under identical exposure and magnification conditions. The values are shown relative to those before treatment. The Western blot gels were cropped and the full-length gels are presented in Supplementary Fig. 6. The data are expressed as the mean ± SD of three replicates each. *P < 0.05 vs. the control.

Thus, to identify the miR-33-5p target region in Hmga2 mRNA, we constructed an Hmga2 3′ UTR luciferase reporter containing mutant sequences of the miR-33-5p binding sites (MUT Hmga2 3′ UTR reporter) and then co-transfected this reporter with miR-33-5p oligos into MC3T3-E1 cells (Fig. 5a). The luciferase reporter assay demonstrated that mimic-33 decreased WT Hmga2 3′ UTR luciferase reporter activity, whereas inhibitor-33 increased WT Hmga2 3′ UTR luciferase reporter activity, but not MUT Hmga2 3′ UTR reporter activity (Fig. 5b). After transfection with miR-33-5p modulators, overexpression of miR-33-5p decreased Hmga2 protein levels, while knockdown of miR-33-5p increased Hmga2 protein levels, though the Hmga2 mRNA level only slightly changed (Fig. 5c,d). Indirect immunofluorescence assays showed the same trends: mimic-33 decreased and inhibitor-33 increased the fluorescence signal of Hmga2 (Fig. 5e).

### Hmga2 levels in MC3T3-E1 cells are altered under different mechanical environments

**Figure 6:**
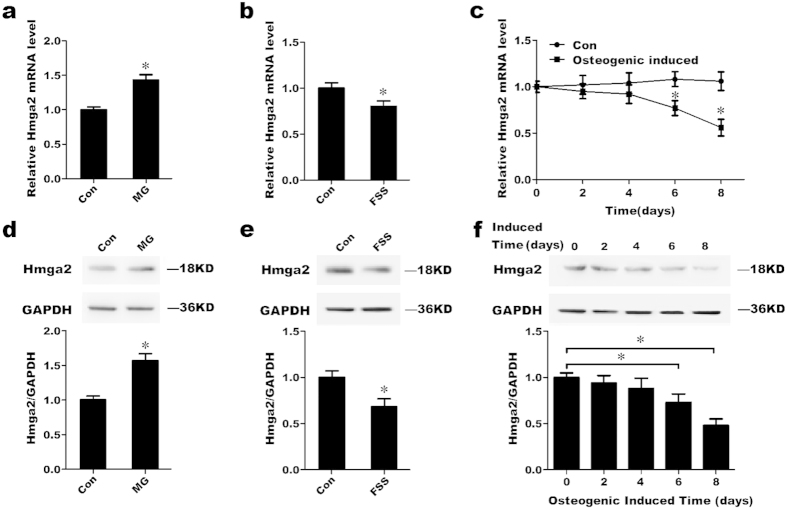
Hmga2 levels in MC3T3-E1 cells are altered under different mechanical environments. (**a**,**b**) qRT-PCR analysis of the changes in the expression of Hmga2 in MC3T3-E1 cells in response to simulated microgravity environment for 48 h or FSS treatment (10 dynes/cm^2^) for 1 h. (**d**,**e**) Western blot analysis of the changes in Hmga2 protein expression in MC3T3-E1 cells in response to simulated microgravity for 48 h or FSS treatment (10 dynes/cm^2^) for 1 h. (**c**,**f**) qRT-PCR and western blot analyses of the expression patterns of Hmga2 mRNA and protein during the differentiation of MC3T3-E1 cells. The values are shown relative to those before treatment. The Western blot gels were cropped and the full-length gels are presented in Supplementary Fig. 6. The data are expressed as the mean ± SD of three replicates each. *P < 0.05 vs. the control.

To further clarify the changes in Hmga2 mRNA and protein expression in response to different mechanical environments, MC3T3-E1 cells were treated with simulated microgravity for 2 days or with 10 dynes/cm^2^ FSS for 1 h. The qRT-PCR analysis showed that the mRNA level of Hmga2 significantly increased in response to simulated microgravity compared with the control group, whereas FSS notably decreased the mRNA expression of Hmga2 (Fig. 6a,b). The protein expression of Hmga2 showed similar trends, presenting a significant increase in response to a simulated microgravity environment and a marked decrease in response to FSS (Fig. 6d,e). We also examined the changes in the relative Hmga2 expression level during osteogenic differentiation over time. Both the mRNA and protein expression levels gradually diminished and were significantly decreased starting on the sixth day after the induction of osteogenic differentiation in MC3T3-E1 cells (Fig. 6c,f).

### Hmga2 inhibits the differentiation of MC3T3-E1 cells

**Figure 7:**
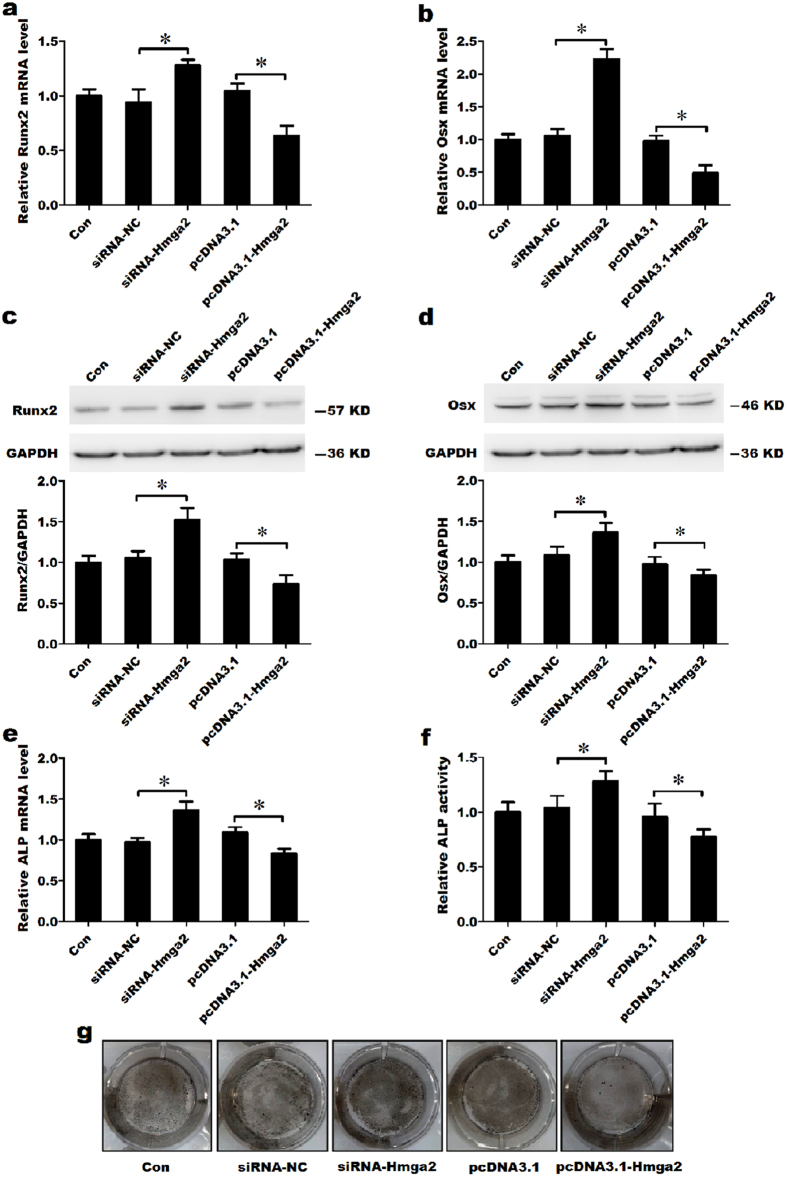
Hmga2 inhibits the differentiation of MC3T3-E1 cells. (**a**,**b**,**e**) qRT-PCR analysis of changes in the mRNA expression of Runx2, Osx and ALP in MC3T3-E1 cells after treatment with siRNA-Hmga2, pcDNA3.1-Hmga2 or their negative controls for 48 h. (**c**,**d**) Western blot analysis of the changes in Runx2 and Osx protein levels in MC3T3-E1 cells after treatment with siRNA-Hmga2, pcDNA3.1-Hmga2 or their negative controls for 48 h. (**f**) The activity analysis of ALP in MC3T3-E1 cells after treatment with siRNA-Hmga2, pcDNA3.1-Hmga2 or their negative controls for 48 h. (**g**) Representative images of ALP staining in MC3T3-E1 cells after treatment with siRNA-Hmga2, pcDNA3.1-Hmga2 or their negative controls for 48 h. The values are shown relative to those before treatment. The Western blot gels were cropped and the full-length gels are presented in Supplementary Fig. 7. The data are expressed as the mean ± SD of three replicates each. *P < 0.05 vs. the control.

To characterize the role of Hmga2 in the osteogenic differentiation of MC3T3-E1 cells, we used expression vector and siRNA to study the effect of gain- and loss-of-function of Hmga2. The qRT-PCR results showed that transfection of siRNA-Hmga2 markedly increased the mRNA expression levels of Runx2, Osx and ALP compared with the transfection of siRNA-NC. Transfection of pcDNA3.1-Hmga2 remarkably decreased the mRNA expression levels of Runx2, Osx and ALP compared with the transfection of blank pcDNA3.1 (Fig. 7a,b,e). This change was also observed at the protein level, as indicated by western blotting, ALP activity assay and ALP staining (Fig. 7c,d,f,g).

### Induction of osteoblast differentiation by miR-33-5p partially depends on Hmga2

**Figure 8:**
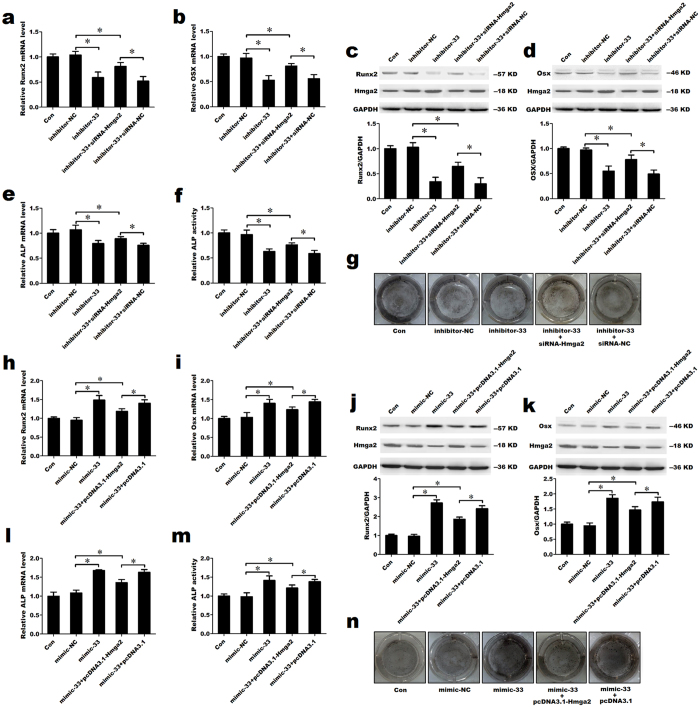
Induction of osteoblast differentiation by miR-33-5p partially depends on Hmga2. (**a**,**b**,**e**) qRT-PCR analysis of the changes in the mRNA expression of Runx2, Osx and ALP in MC3T3-E1 cells after the co-transfection of inhibitor-33 and siRNA-Hmga2 or its negative control. (**c**,**d**) Western blot analysis of the changes in Runx2, Osx and Hmga2 protein levels in MC3T3-E1 cells after co-transfection of inhibitor-33 and siRNA-Hmga2 or its negative control. (**f**) The activity analysis of ALP in MC3T3-E1 cells after the co-transfection of inhibitor-33 and siRNA-Hmga2 or its negative control. (**g**) Representative images of ALP staining in MC3T3-E1 cells after the co-transfection of inhibirot-33 and siRNA-Hmga2 or its negative control. (**h**,**i**,**l**) qRT-PCR analysis of the changes in the mRNA expression of Runx2, Osx and ALP in MC3T3-E1 cells after the co-transfection of mimic-33 and pcDNA3.1-Hmga2 or its negative control. (**j**,**k**) Western blot analysis of the changes in Runx2, Osx and Hmga2 protein levels in MC3T3-E1 cells after co-transfection of mimic-33 and pcDNA3.1-Hmga2 or its negative control. (**m**) The activity analysis of ALP in MC3T3-E1 cells after the co-transfection of mimic-33 and pcDNA3.1-Hmga2 or its negative control. (**n**) Representative images of ALP staining in MC3T3-E1 cells after the co-transfection of mimic-33 and pcDNA3.1-Hmga2 or its negative control. The values are shown relative to those before treatment. The Western blot gels were cropped and the full-length gels are presented in Supplementary Fig. 7. The data are expressed as the mean ± SD of three replicates each. *P < 0.05 vs. the control.

To further confirm that the induction of osteoblast differentiation by miR-33-5p depends on Hmga2, we co-transfected inhibitor-33 with siRNA-Hmga2 or its negative control. The co-transfection of inhibitor-33 with siRNA-Hmga2 partially blocked the inhibitor-33-induced reduction of Runx2, Osx and ALP at the mRNA level, as indicated by qRT-PCR (Fig. 8a,b,e). These changes persisted at the protein level, where co-transfection of inhibitor-33 with siRNA-Hmga2 partially blocked the reduction of Runx2 and Osx proteins levels, the decrease of ALP activity and ALP staining induced by inhibitor-33 (Fig. 8c,d,f,g). Moreover, we co-transfected mimic-33 with pcDNA3.1-Hmga2 or blank pcDNA3.1. The co-transfection of mimic-33 with pcDNA3.1-Hmga2 partially inhibited the mimic-33-induced increases of Runx2, Osx and ALP at the mRNA level (Fig. 8h,I,l). And the similar alterations were observed at the protein level, where co-transfection of mimic-33 with pcDNA3.1-Hmga2 partially blocked the increases of Runx2 and Osx proteins levels, ALP activity and ALP staining induced by mimic-33 (Fig. 8j,k,m,n).

## Discussion

A recent study demonstrated that miRNAs respond to mechanical loading and play essential roles in osteoblast differentiation and bone formation^28^. In the present study, we identified miR-33-5p as a novel mechano-sensitive miRNA that positively regulates osteoblast differentiation by repressing Hmga2 expression at the post-transcriptional level. Moreover, we demonstrated negative regulation of Hmga2 in osteoblast differentiation. Thus, miR-33-5p is a potential therapeutic target for the treatment of pathological disorders of skeletal development induced by changes in the mechanical environment. This study shows, for the first time to our knowledge, that a miRNA is sensitive to two essentially different mechanical stimuli: FSS, a contact force, and gravity, a non-contact force.

Mechanical stretching has previously been identified as an important regulator of diverse biological and pathological processes, and FSS, a common and potent type of mechanical stimulation in bone, has been found to play an important role in osteoblast differentiation, development and function^29^,^30^. Previous studies have identified many important pathways and molecules that participate in osteoblast differentiation induced by FSS. FSS modulates the activity of Akt, GSK-3b, and β-catenin, which are important signaling components of the mechanotransduction pathway in osteoblasts that promote cellular differentiation^31^,^32^. The increase in IL-11 expression via the binding of the ΔFosB/JunD complex to the 5′AP-1 site of the IL-11 gene promoter plays an important role in osteoblast differentiation stimulated by FSS, and increases in IL-11 enhance osteoblastogenesis by stimulating Wnt/β-catenin signaling^7^,^12^. Additionally, the Cbfa1/Runx2, MAPK, NO/cGMP/PKG and Ca^2+^ signaling pathways have been shown to be responsive to FSS^11^,^33^. Gravity also plays an irreplaceable role in osteoblast differentiation. For example, miR-214 inhibits osteoblast differentiation and activity by directly targeting ATF4 in hind limb-unloaded mice^34^, and miR-153 down-regulates osteoblast differentiation in hMSCs by directly targeting BMPR2 in a microgravity environment^24^. Microgravity also affects the post-translational modification of collagen by altering the expression of enzymes involved in the formation of cross-links^3^,^35^. Moreover, the ERK/MAPK, canonical Wnt and TNF-α signaling pathways have been found to be involved in the regulation of osteoblast differentiation and function in a microgravity environment^36^,^37^,^38^. Although many studies have examined the mechanisms by which FSS or microgravity regulate osteoblast differentiation, these studies did not identify a common mechanism through which two essentially different types of mechanical stimulation (i.e., a contact force and a non-contact force) regulate osteoblast differentiation. To our knowledge, this study is the first to investigate the molecular mechanism of mechanotransduction in different multiple mechanical environments. Specifically, we found that the expression of miR-33-5p in MC3T3-E1 cells increased in response to FSS and decreased in response to a simulated microgravity environment. Thus, miR-33-5p is the first mechano-sensitive miRNA to be identified as sensitive to both contact forces (FSS) and non-contact forces (gravity).

The miR-33 miRNA family is highly conserved from Drosophila to humans. Two isoforms of miR-33 are expressed in humans, miR-33a and miR-33b. However, only one miR-33 isoform is expressed in mice and conserved in humans, miR-33a. Human miR-33a features two subtypes, miR-33a-3p and miR-33a-5p, which correspond to miR-33-3p and miR-33-5p in mice, respectively^39^,^40^. In 2007, the down-regulation of neural genes by miR-33 was reported to be attenuated by down-regulation of FoxG1 expression during forebrain development^41^. Subsequent studies demonstrated that miR-33a, an intronic microRNA located within the SREBF-2 gene, plays an important role in the homeostatic regulation of cholesterol metabolism^42^,^43^. Furthermore, miR-33a expression in both macrophages and hepatocytes has been shown to be inversely correlated with the level of cholesterol^44^,^45^. Knockdown of miR-33 also promotes cholesterol trafficking *in vitro* and high-density lipoprotein (HDL) synthesis *in vivo*^46^. Many studies subsequently performed antisense therapeutic targeting of miR-33 in individuals suffering from cardiometabolic diseases^47^,^48^,^49^,^50^,^51^. In addition to the relationship of miR-33 with cholesterol metabolism, this miRNA is up-regulated in human papillomavirus-positive cases of squamous cell carcinoma of the head and neck^52^ and down-regulated in biopsies from myotonic dystrophy type-1 patients^53^. Furthermore, one study indicated that overexpression of miR-33a in A375 cells significantly inhibited melanoma tumorigenesis, identifying miR-33 as a tumor suppressor in melanoma^54^. Moreover, miR-33 has recently been found to be able to regulate critical genes involved in cellular energy sensing (AMPK)^55^, mitochondrial biogenesis, and mitochondrial fatty acid oxidation (CROT, CPT1a, HADHB). The present results extend these earlier findings by demonstrating that miR-33-5p also regulates osteoblast differentiation in MC3T3-E1 cells. Specifically, our data show that overexpression of miR-33-5p increases the expression of canonical biomarkers of osteoblast differentiation (i.e., Runx2 and Osx), indicating that miR-33-5p promotes osteoblast differentiation *in vitro*. Moreover, the ability of miR-33-5p to promote osteoblast differentiation was verified in simulated microgravity and FSS environments. The overexpression of miR-33-5p partially attenuated the inhibition of osteoblast differentiation caused by simulated microgravity, which indicated that gravity regulates osteoblast differentiation via miR-33-5p. Accordingly, our results show that miR-33-5p participates in the regulation of osteoblast differentiation by FSS, and knockdown of this miRNA partially inhibits this differentiation. These data indicate that miR-33-5p may act as a common sensor of mechanotransduction in different multiple mechanical environments.

Some aspects of the mechanisms by which miR-33 regulates gene expression are already known. Specifically, miR-33a/b targets adenosine triphosphate–binding cassette transporter A1 (ABCA1) to regulate HDL synthesis and reverse cholesterol transport^42^,^44^. In mouse macrophages, miR-33 also targets adenosine triphosphate–binding cassette transporter G1 (ABCG1), reducing cholesterol efflux to nascent HDL^56^. Moreover, miR-33 has been shown to mediate the down-regulation of p53, a process that depends on the binding of miR-33 to two conserved motifs in the 3′ UTR of p53 that control hematopoietic stem cell self-renewal^57^. miR-33 also targets the RIP140 mRNA 3′ UTR to reduce the RIP140 co-activator activity for NF-kappaB, which is supported by the reduction of NF-kappaB reporter activity and the inflammatory potential in macrophages^58^. Furthermore, HIF-1alpha was identified as a direct target gene of miR-33a^54^. Crucially, Hmga2 was found to be a target of miR-33a in mouse and human lung cancer cells^59^. It was also found to be a target of miR-33b in human breast cancer cells^60^. In this study, we confirmed that Hmga2 is a target gene of miR-33-5p in mouse pre-osteoblasts, MC3T3-E1 cells, as indicated by a luciferase assay. Furthermore, miR-33-5p negatively regulated Hmga2 expression at the post-transcriptional level.

The high mobility group AT-hook (HMGA) proteins, a family of DNA architectural factors, are highly expressed during embryogenesis and play crucial roles in several different biological processes and in the tumorigenesis of a wide range of tissues, including pituitary tumors^27^. Many studies have confirmed that Hmga2 plays a critical role in the development of both benign and malignant neoplasias, including carcinomas of the pancreas, thyroid, colon, breast, lung, ovary, and prostate, squamous carcinomas of the oral cavity, and head and neck tumors^61^,^62^. In addition to its critical effect on tumors, Hmga2 is involved in a wide spectrum of biological processes, including embryonic development, cell differentiation and transformation, cell cycle progression, apoptosis, senescence, DNA repair, and various cellular pathologies^63^. However, the role of Hmga2 in the skeletal system is poorly understood. Constitutional rearrangement of Hmga2 in an 8-year old boy was shown to result in extreme overgrowth and advanced bone development^64^. A large population-based study found that a common genetic variant of Hmga2 is intimately related to both the trabecular bone mineralization density (BMD) in ethnically diverse older men and variations in height in the general population^65^,^66^. In addition, Hmga2 mRNA and protein are expressed in the human fetal osteoblast cell line hFOB, but not other human osteoblast cell lines, such as MG63, SaOS-2, HOS, and U2-OS^66^. Furthermore, as the target gene of let-7, Hmga2 was confirmed to suppress osteogenesis of human mesenchymal stem cells^67^. In this study, Hmga2 was found to negatively regulate osteoblast differentiation in MC3T3-E1 cells. Moreover, the regulatory effect of miR-33-5p was shown to partially depend on Hmga2, and Hmga2 was identified as a downstream regulator of miR-33-5p that participates in the mechanism by which miR-33-5p regulates osteoblast differentiation. This finding revealed a new function for Hmga2 in addition to its important role in tumors and indicated that the function of Hmga2 warrants further study.

Notably, this study was also subject to limitations. Based on our previous research^68^, we investigated the effect of 10 dynes/cm^2^ FSS for 1 h on osteoblast differentiation. However, the magnitudes and application time of FSS may affect osteoblast differentiation^69^. Thus, the role of miR-33-5p in FSS-induced osteoblast differentiation needs to be studied in several different FSS environments. Moreover, the *in vitro* results obtained using MC3T3-E1 cells in this study have not yet been confirmed *in vivo*. Accordingly, we plan to confirm our findings *in vivo* in a future study.

In conclusion, our study showed, for the first time, that a specific mechano-sensitive miRNA, miR-33-5p, senses multiple mechanical environments in osteoblasts and subsequently modulates osteoblast differentiation in response to both contact and non-contact forces *in vitro*. Specifically, miR-33-5p functions by inhibiting its direct target, Hmga2, at the post-transcriptional level to negatively affect osteoblast differentiation. These findings not only provide new insights into mechanotransduction in osteoblasts but also suggest that miRNAs can be targeted in bone tissue engineering for regenerative medicine applications. We expect miR-33-5p to be targeted for the treatment of human bone remodeling disorders that are related to mechanical loading, such as osteoporosis or osteopenia induced by microgravity.

## Method

### Cell culture and transfection

MC3T3-E1 cells (P8–P12), a mouse osteoblast-like cell line, were grown in DMEM (Hyclone, USA) containing 10% heat-inactivated fetal bovine serum (Hyclone, USA), 100 U/ml penicillin G, and 100 mg/ml streptomycin. The cells were maintained in a humidified incubator at 37 °C under 5% CO_2_ and subcultured every 72 h. For all the osteogenic differentiation experiments, cells were induced by culturing in osteogenic medium (OM; 10% FBS, 0.1 mM dexamethasone, 10 mM b-glycerophosphate, and 50 μg/mL ascorbic acid in DMEM).

To transfect cells with miRNA regulators, siRNA oligos or plasmid, the medium was supplemented with Lipofectamine™ 2000 (Invitrogen, USA), which was used according to the manufacturer’s instructions. A miR-33 mimic or inhibitor (RiboBio, China) was transfected at a concentration of 100 nM. A siRNA targeting Hmga2 (GenBank Accession NM_010441) was designed. The sequences of siRNA and its negative control are listed in Supplementary Table 1. The expression vector of Hmga2, pcDNA3.1-Hmga2 WT, was obtained from non-profit repository AddGene (Plasmid #14789). The siRNA was transfected at a concentration of 80 nM and the plasmid was transfected at a concentration of 200 ng/μl.

### Fluid shear stress

Fluid flow was generated by parallel plate flow chambers with gravity-driven fluid flow using a peristaltic pump (Ibidi, German). By adjusting the channel height and flow rate, stress levels of 10 dynes/cm^2^ were generated^32^,^68^. The cells were plated in a flow chamber with a surface area of 2.5 cm^2^. Control cells were plated in the same chambers but were not subjected to FSS. Each test was conducted for the indicated time. The entire flow system was encased within a large incubator at 37 °C.

### Clinorotation to simulate microgravity

Due to the limitations of real spaceflight, most studies on the biological effects of microgravity are conducted using ground-based analogs. A clinostat is an effective, ground-based tool for simulating microgravity. A clinostat consists of a vertical turntable and a horizontal turntable. The vertical chambers rotate around the horizontal axis, which designates clinorotation. Clinorotation mimics certain aspects of the microgravity environment by nullifying the integrated gravitational vector via continuous averaging. The horizontal chambers rotate around the vertical axis, which acts as a rotational control. In this study, the cells were exposed to clinorotation for 48 h at 24 rpm^25^. Specifically, the cells were seeded at a density of 1 × 10^5 ^cells on 2.5 cm × 3.0 cm coverslips, which were placed in 6-well plates. After the cells grew for 24 h and adhered to the coverslips, the coverslips were inserted into the fixtures of the chambers, which were subsequently completely filled with DMEM containing 10% FBS and aspirated to eliminate the presence of air bubbles. The chambers were divided into two groups: the horizontal rotation control and clinorotation groups. The clinostat was maintained in an incubator at 37 °C.

### Taqman miRNA assay and qRT-PCR

Total RNA from bone tissues or cells was extracted with the TRIzol Reagent (Invitrogen, USA) according to the manufacturer’s instructions. First-strand cDNA was synthesized by incubating 1 μg of total RNA with Superscript III reverse transcriptase (Takara, Japan) for 1 h at 42 °C following oligo (dT) priming. After reverse transcription, qRT-PCR was performed using a CFX96 (BIO-RAD, USA) instrument and SYBR Premix Ex TaqTM (Takara, Japan) according to the manufacturer’s instructions. All amplifications were normalized against GAPDH. The data were analyzed via the relative Ct (ΔΔCt) method and were expressed as a fold change compared with the respective control. Each sample was analyzed in triplicate. The sequences of primers used for qRT-PCR are contained in Supplementary Table 1.

The miRNA was reverse transcribed using the TaqMan miRNA reverse transcription kit (Sangon Biotech, China) and miRNA-specific primers designed by Primer Premier 5.0 software. miRNA expression levels were then analyzed using the TaqMan miRNA assay Kit (Sangon Biotech, China) according to the manufacturer’s instructions. Quantitation of the ubiquitously expressed miRNA, U6, was performed as an endogenous control. Primer sequences are provided in Supplementary Table 2.

To verify the miRNA quantification in another method, we used qRT-PCR to test the changes of miRNAs. The PrimeScript® RT reagent kit (Takara, Japan) was used to synthesize cDNA, but the Oligo dT Primer and Random 6 mers in the kit were replaced with stem-loop miRNA RT primers. Subsequent qRT-PCR detection was performed under the same conditions used for mRNA detection, as described above. U6 small nuclear RNA was used as an internal control. The sequences of primers are listed in Supplementary Table 1.

### Western blot analysis

Cells were lysed using RIPA buffer (Thermo scientific, USA) containing a protease inhibitor cocktail (Roche, Switzerland). Equal amounts of protein from each sample were added to a NuPage Bis-Tris polyacrylamide gel (Invitrogen, USA) and run for 2 h using MES SDS running buffer (Invitrogen, USA). The proteins were then transferred to nitrocellulose membranes, which were blocked for 5 h at room temperature with milk (5% w/v) in Tris-buffered saline (TBS) containing Tween-20 (0.1%; TBS-T). The blots were subsequently incubated overnight with a primary antibody (1: 2,000) against Runx2 or Osx at 4 °C with oscillation, after which they were incubated with a horseradish peroxidase-conjugated secondary antibody (1: 10,000; Jackson, USA). The secondary antibodies were detected and visualized using the Super Signal West substrate (Fisher Scientific, USA). The resultant bands were quantified through densitometry with Image J software. The information of antibodies in detail showed as follows. Runx2 antibody (Abcam, ab23981, USA); Osx antibody (Abcam, ab22552, USA); Hmga2 antibody (Cell signal technology, #8179, USA).

### Alkaline Phosphatase Activity Assay

MC3T3-E1 cells were rinsed two times with ice-cold PBS, lysed with 0.1 M M-PER mammalian protein extraction reagent (Pierce, USA) for 30 minutes, and finally centrifuged at 10,000 g for 15 minutes. ALP activity of the supernatants was determined at 405 nm using p-nitrophenyl phosphate (pNPP) (Sigma Aldrich, USA) as the substrate. A 50 μl of sample was mixed with 50 μl of pNPP (1 mg/ml) in 1 M diethanolamine buffer containing 0.5 mM MgCl_2_ (pH 9.8) and incubated at 37 °C for 15 mins on a bench shaker. The reaction was stopped by the addition of 200 μl of 2 M NaOH per 200 μl of reaction mixture. Total protein content was determined by the BCA method with protein assay kit (Pierce, USA). Calf intestinal alkaline phosphatase (Sigma Aldrich, USA) was used as the standard. One unit of the standard will hydrolyze 1 μmol of pNPP per min at 37 °C.

### Alkaline Phosphatase staining

ALP staining was typically performed using the ALP staining kit (SenBeiJia Biological Technology, Nanjing, China) according to the manufacturer’s instructions. All the experiments were repeated triply. And the representative images were taken by digital camera.

### Immunofluorescence

Cell preparation and nuclear staining. Cultured osteoclasts were gently washed with fresh media to remove nonadherent cells before fixation in 2.0% paraformaldehyde for 15 min at room temperature. Cells were then permeabilized in 0.025% Triton X-100 in PBS for 10 min and incubate cells for 1 hour with normal goat serum. After introduced diluted primary antibody to the cells overnight at 2–8 °C, incubated the cells with secondary antibody-FITC (Abcam, ab7086, USA) diluted in blocking solution in a dark humidity chamber for 1 hour. Counterstain sample with DAPI at room temperature for 10 minutes, and store the slides in the dark at 2–8 °C. The micrographs were shot by laser scanning confocal microscope (Olympus FV1000, Japan).

### Luciferase assay

Using Lipofectamine 2000 (Invitrogen, USA), MC3T3-E1 cells grown in a 96-well plate were co-transfected with 150 ng of either empty vector or miR-33, 50 ng of a firefly luciferase reporter including the 3′ UTR of Hmga2, and 2 ng of pRL-TK (Promega, Madison, USA). The mute 3′ UTR of Hmga2 acted as a negative control. Cells were harvested for the luciferase assay 48 h after transfection using a luciferase assay kit (Promega, USA) according to the manufacturer’s protocol. Transfection was repeated in triplicate.

### Statistical analysis

The experimental data were statistically analyzed with the SPSS 17.0 software. The data are expressed as the mean ± SD of at least three independent experiments. A repeated-measures one-way ANOVA was used to compare the time-course variables. Comparisons were performed using a two-tailed t-test, or one-way ANOVA for experiments with more than two subgroups. A *P* value of less than 0.05 was considered to be significant.

## Additional Information

**How to cite this article**: Wang, H. *et al.* miR-33-5p, a novel mechano-sensitive microRNA promotes osteoblast differentiation by targeting Hmga2. *Sci. Rep.* **6**, 23170; doi: 10.1038/srep23170 (2016).

## Supplementary Material

Supplementary Information
