# Supplementary material for: miR-33-5p, a novel mechano-sensitive microRNA promotes osteoblast differentiation by targeting Hmga2
Source: Sci Rep. 2016 Mar 16;6:23170. doi: 10.1038/srep23170 (PMC4793269; doi:10.1038/srep23170)
Supplement: Supplementary Information [file srep23170-s1.doc]

**Supplemental information**

miR-33-5p, a novel mechno-sensitive microRNA promote osteoblast differentiation by targeting Hmga2.

Han Wang^1*^, Zhongyang Sun^1,2*^, Yixuan Wang^1*^, Zebing Hu^1^, Hua Zhou^1^, Lianchang Zhang^1^, Bo Hong^1^, Shu Zhang^1^, Xinsheng Cao^1^

**Supplementary Figure 1**

**
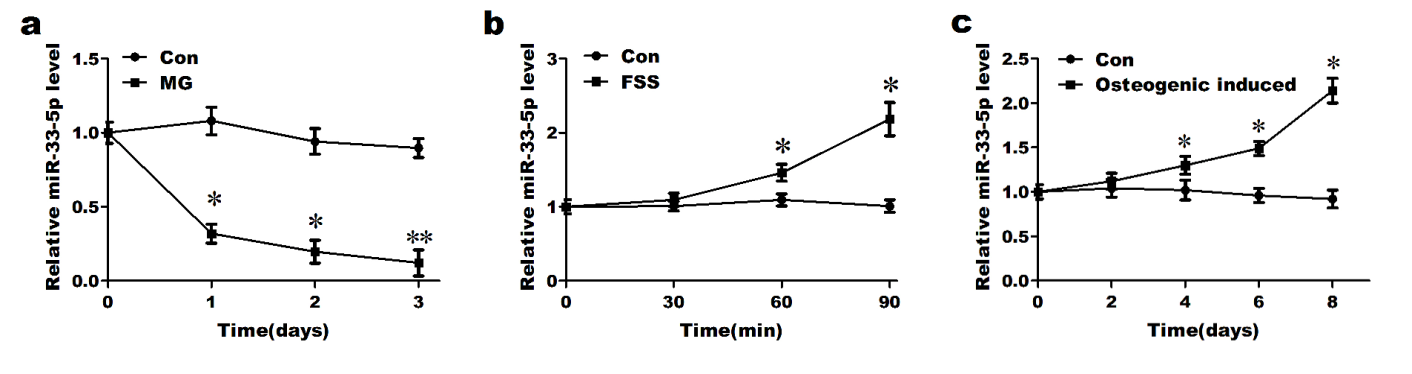
**

**Supplementary Figure 1. The time course of changes of miR-33-5p in MC3T3-E1 cells analyzed by qRT-PCR.** (a) qRT-PCR analysis of the expression pattern of miR-33-5p in MC3T3-E1 cells in response to simulated microgravity for 3 d. (b) qRT-PCR analysis of the expression pattern of miR-33-5p in MC3T3-E1 cells treated with FSS (10 dynes/cm^2^) for 90 min. (c) qRT-PCR analysis of the expression pattern of miR-33-5p during the differentiation of MC3T3-E1 cells. The data are expressed as the mean ± SD of three replicates each. **P* < 0.05, ***P* < 0.01 vs. the control.

**Supplementary Figure 2**

**
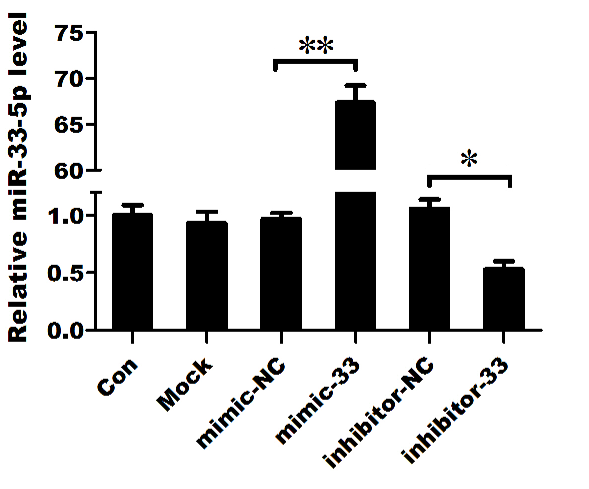
**

**Supplementary Figure 2. The regulative effects of miR-33-5p mimic and inhibitor on miR-33-5p level.** qRT-PCR analysis of changes in the expression of miR-33-5p in MC3T3-E1 cells after treatment with mimic-33, inhibitor-33 or their negative controls (100 nM) for 48 h. The data are expressed as the mean ± SD of three replicates each. **P* < 0.05, ***P* < 0.01 vs. the control.

**Supplementary Figure 3**


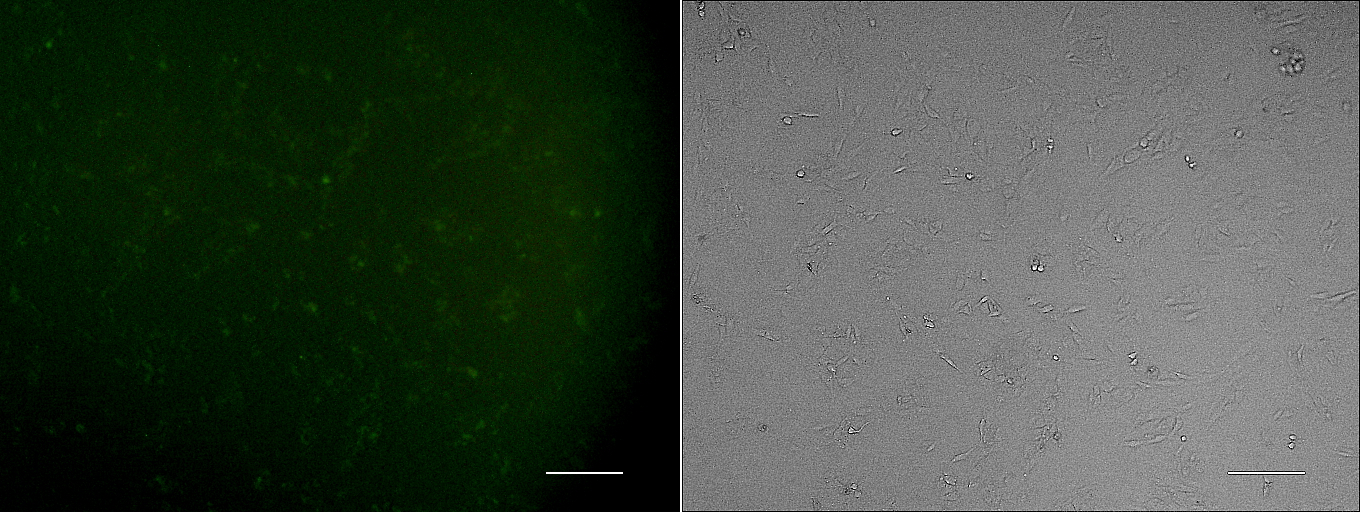


**Supplementary Figure 3. Flurescent image of MC3T3-E1 cells transfected with FAM conjugated miRNA nucleoside analogue.** MC3T3-E1 cells were transfected with a 100 nM concentration of FAM conjugated miRNA nucleoside analogue. After a 24-hour transfection period, microphotographs were taken. The left image show fluorescence phenomenon in dark field. The right image represent the corresponding cells in bright field. Scale bars, 200 μm.

**Supplementary Figure 4**


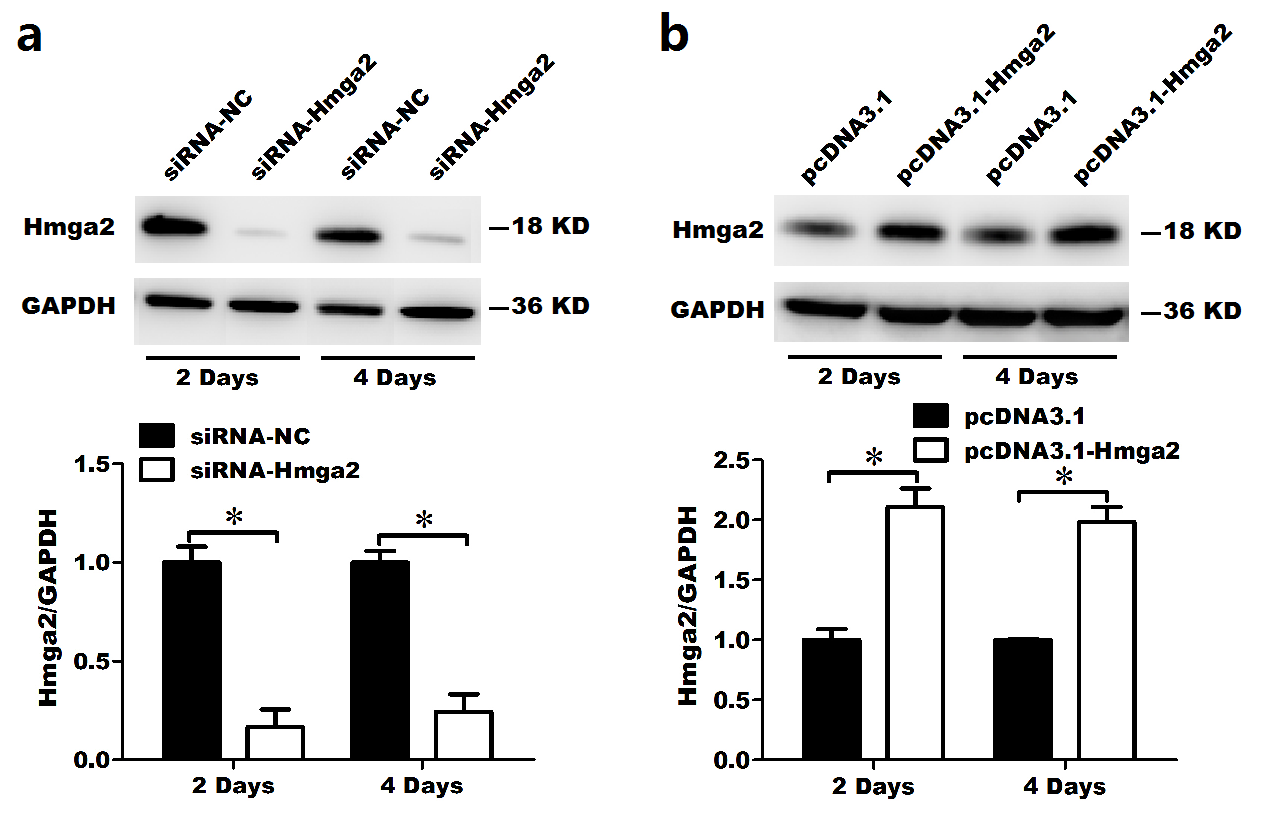


**Supplementary Figure 4. The regulative effect of siRNA-Hmga2 and pcDNA3.1-Hmga2 on Hmga2 protein expression.** (a) Western blot analysis of the changes in Hmga2 protein expression in MC3T3-E1 cells after transfection with siRNA-Hmga2 or its negative control for 2 or 4 d. (80 nM). (b) Western blot analysis of the changes in Hmga2 protein expression in MC3T3-E1 cells after transfection with pcDNA3.1-Hmga2 or pcDNA3.1 for 2 or 4 d. (200 ng/μl). The data are expressed as the mean ± SD of three replicates each. **P* < 0.05, vs. each control.

**Supplementary Figure 5**

**
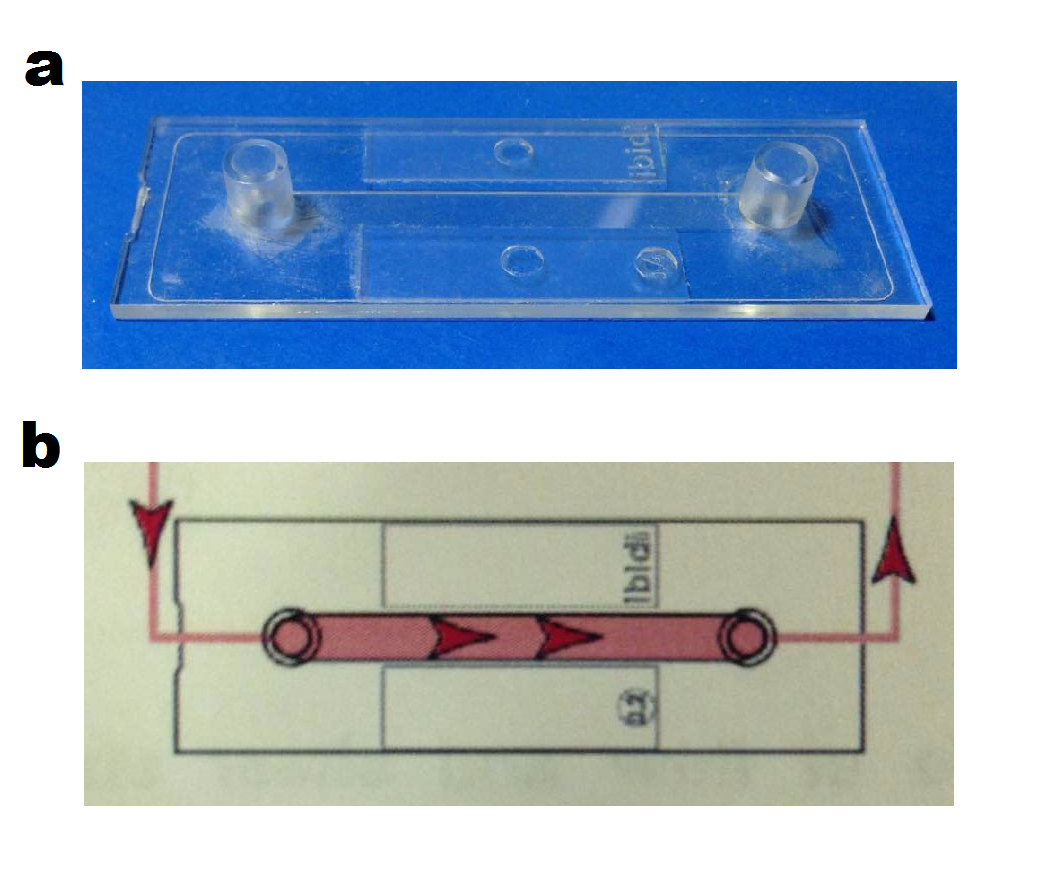
**

**Supplementary Figure 5. Detailed information of the culture slide used in FSS experiments.** (a) The photograph of the FSS culture slide. (b) The schematic illustration of the FSS culture slide.

**Supplementary Figure 6**

**
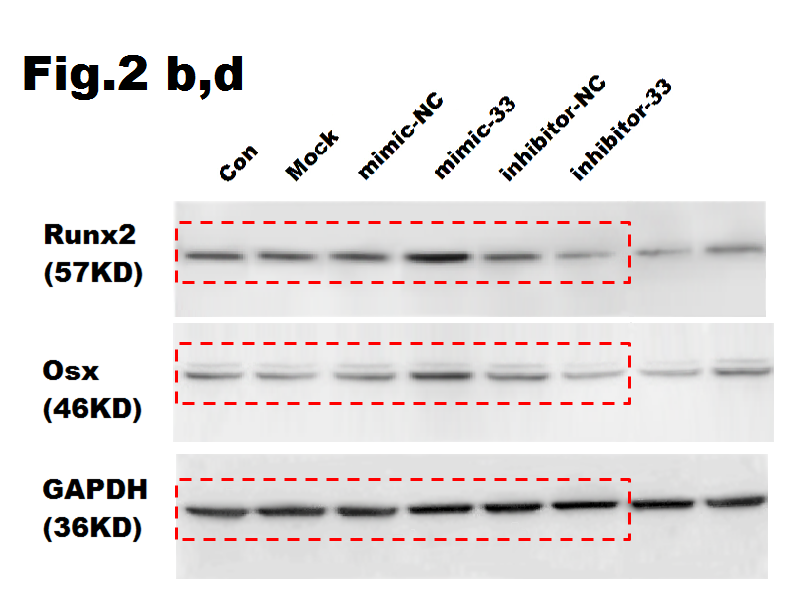

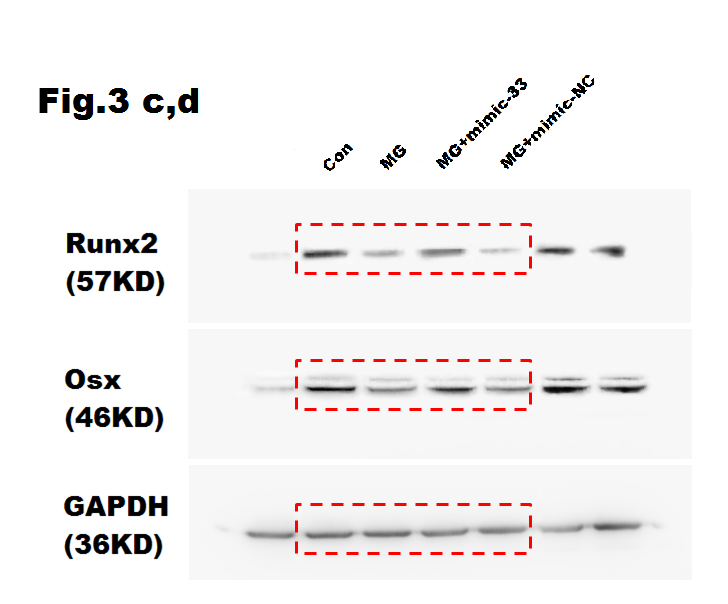
**

**
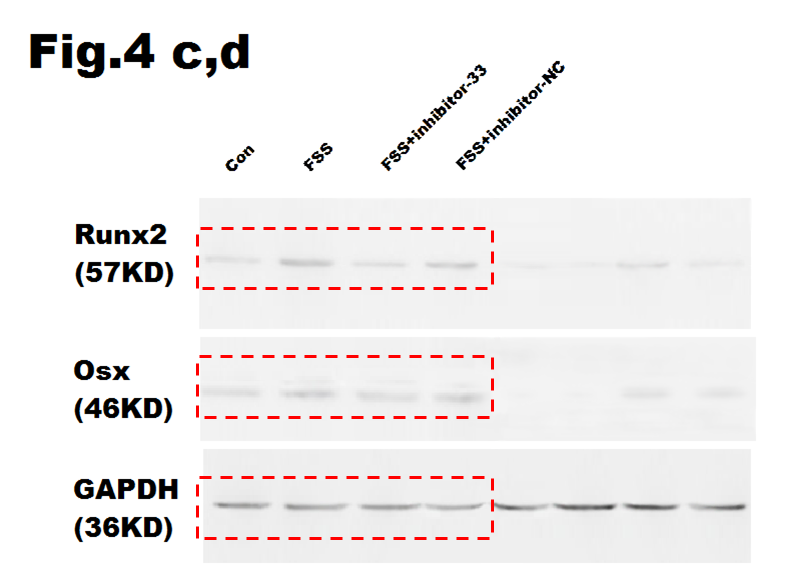

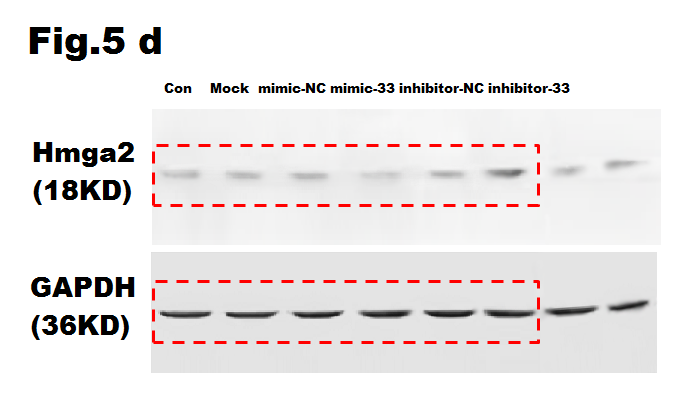
**

**
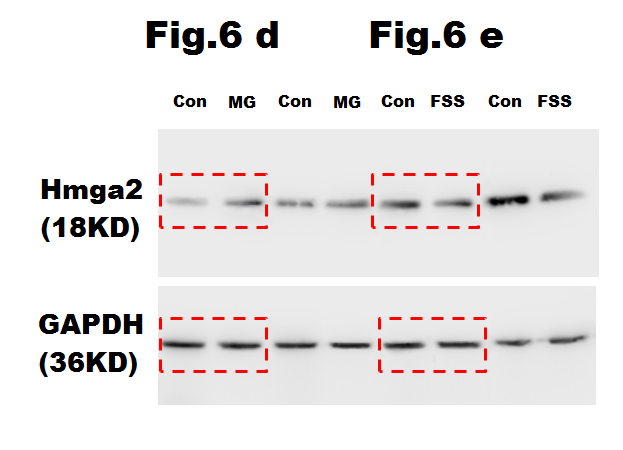

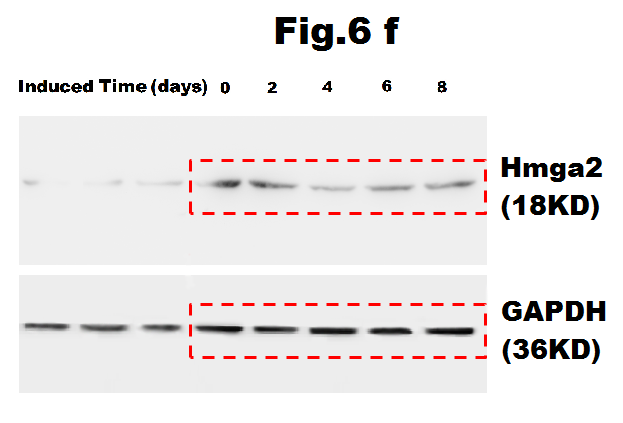
**

**Supplementary Figure 6. Uncropped, unprocessed images of blots and gels.**

**Supplementary Figure 7**

**
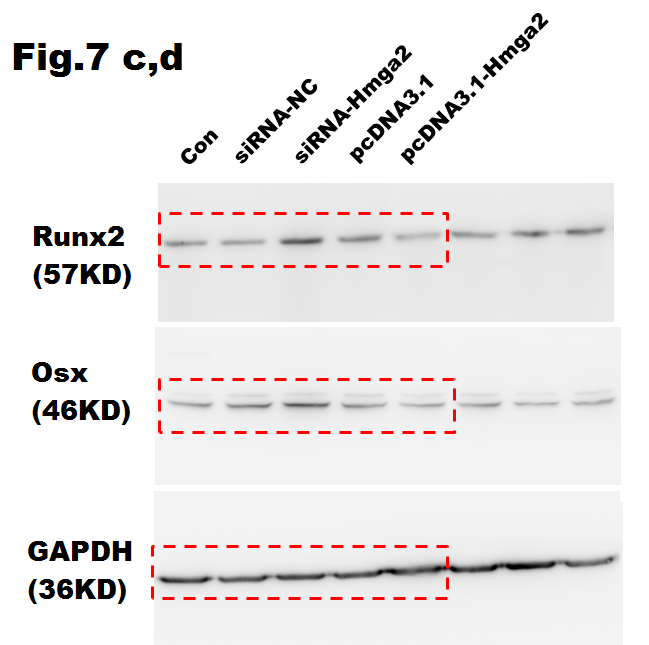
**
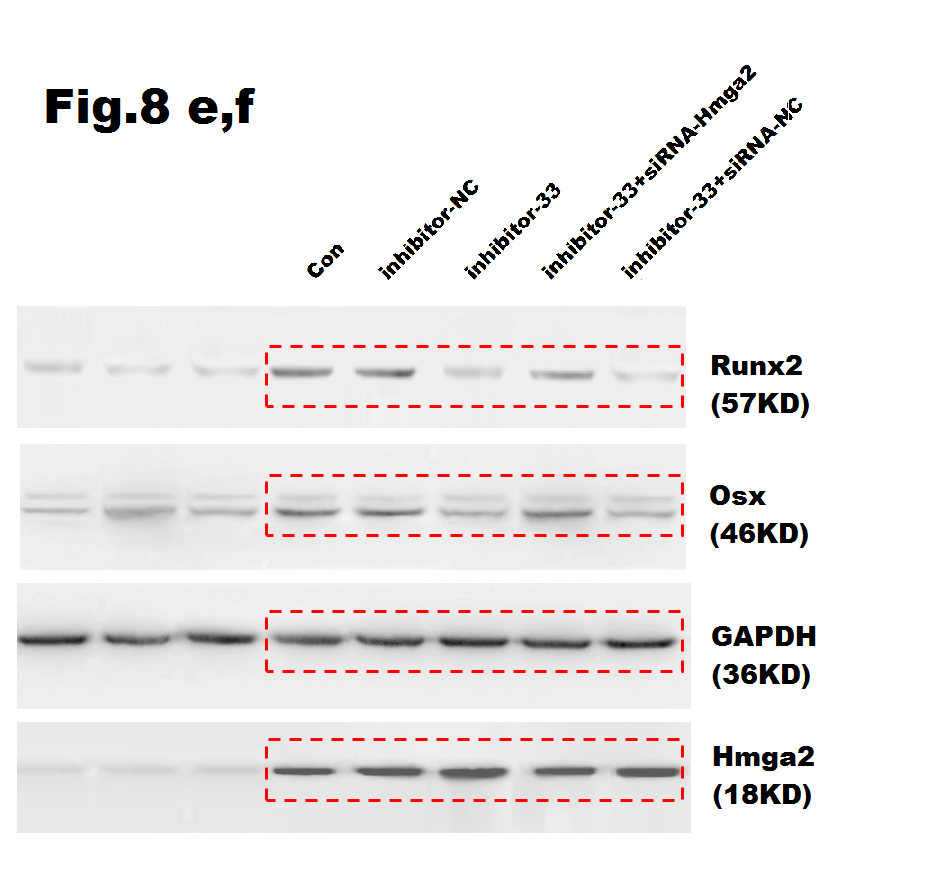


**
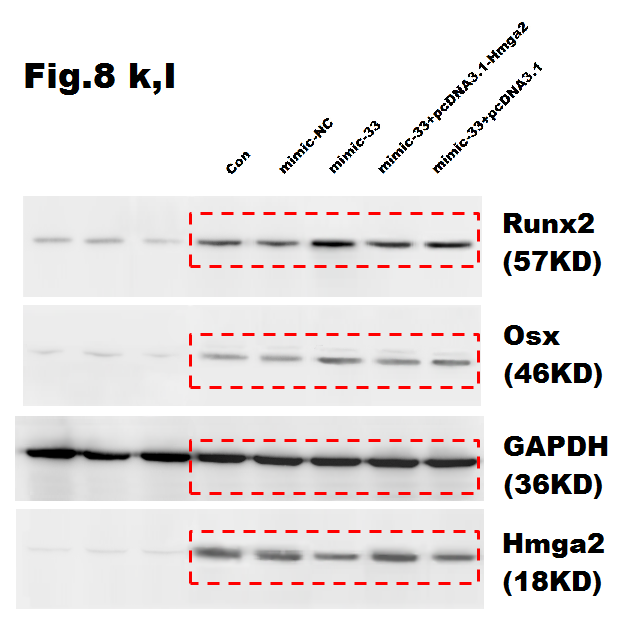
**

**Supplementary Figure 7. Uncropped, unprocessed images of blots and gels.**

**Supplementary Table 1**

**Supplementary Table 1. The sequence of primers and siRNAs.**


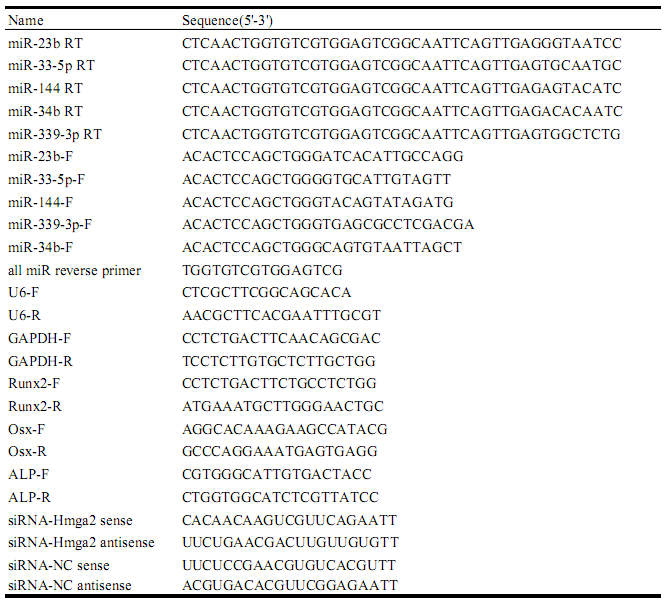


**Supplementary Table 2**

**Supplementary Table 2. The sequence of primers used for TaqMan miRNA assay.**

**
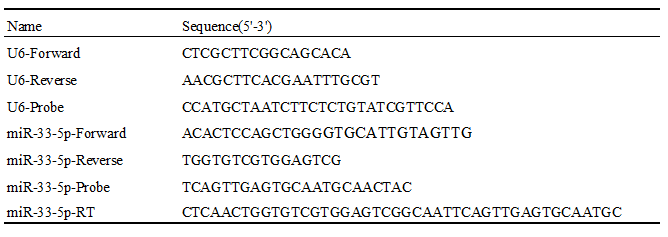
**
